# Trap-related injuries in coypus (*Myocastor coypus*) and raccoons (*Procyon lotor*)—an approach to improve animal welfare in live trapping

**DOI:** 10.3389/fvets.2025.1752107

**Published:** 2026-01-23

**Authors:** Friederike Gethöffer, Franziska M. Schöttes, Maximilian Reuschel, Peter Wohlsein, Andreas Beineke, Ursula Siebert

**Affiliations:** 1 Institute for Terrestrial and Aquatic Wildlife Research, University of Veterinary Medicine Hannover, Hannover, Germany; 2 Department of Small Mammal, Reptile and Avian Medicine and Surgery, University of Veterinary Medicine Hannover, Hannover, Germany; 3 Department of Pathology, University of Veterinary Medicine Hannover, Hannover, Germany

**Keywords:** animal welfare, injuries, invasive species, live trap, *Myocastor coypus*, *Procyon lotor*, wildlife management

## Abstract

Live trapping is a common method in wildlife research and management, yet it poses inherent risks to animal welfare. This study systematically evaluated injury incidence and severity in coypus (*Myocastor coypus*) and raccoons (*Procyon lotor*) captured using three commercially available live trap types: a standard wooden box trap (WBT), a metallic, sheet metal trap (SMT), and a wire grid trap (WGT). A total of 55 coypus and 45 raccoons were examined following a trap confinement duration of a maximum of six hours. Injuries were assessed using standardized necropsy protocols and categorized by anatomical location, severity, and presumed cause. Results showed species-specific injury patterns, with raccoons exhibiting more frequent and severe injuries than coypus. Raccoons primarily sustained skin lesions and dental trauma, consistent with their manipulative, escape-oriented behavior, while coypu injuries were predominantly localized to the snout and incisors. Although not statistically significant, trap design influenced injury profiles: the WBT was associated with severe injuries in raccoons, particularly to the forelimbs and dentition, while the WGT prompted intense escape behaviors without a proportional increase in trauma. The SMT resulted in fewer external injuries but did present species-specific risks, such as claw-abrasion and tail entrapment. Approximately 93% of raccoons and 55% of coypus exhibited external injuries, including 14 severe cases and 5 confirmed fractures. These findings underscore the importance of species-specific trap assessment and design optimization to mitigate animal suffering. Given the limitations of traditional injury scoring systems and behavioral indicators when applied to wild animals, this study highlights the need for integrated, evidence-based welfare assessments in field settings. Future research should prioritize refinement of trapping methods and standardized welfare evaluation frameworks to support ethical and effective wildlife management.

## 1 Introduction

In recent decades, wild animal welfare has become an increasingly important topic in public and scientific discourse, e.g., (1). Live trapping methods, in particular, have been the subject of critical debate, often raising concerns regarding their acceptability under animal welfare standards (2–4). This scrutiny has led to political pressure on authorities and practitioners, reinforcing the need for more comprehensive research to evaluate the animal welfare implications of live trapping (5–7).

Live trapping is a widespread method employed in Germany for managing, e.g., invasive alien species (IAS). Within the European Union, the management of invasive species such as coypu (*Myocastor coypus*) and raccoon (*Procyon lotor*) is governed by Regulation (EU) 1143/2014, which mandates a three-phase management framework for listed invasive alien species. In Germany, both species are subject to the Federal Hunting Act (BJagdG) and are regulated at the state level through respective hunting laws, such as the Hunting Act of Lower Saxony (NJagdG), which prescribes seasonal hunting. According to surveys among hunting practitioners in Lower Saxony, trapping accounts for 44–74% of all coypu and raccoon removals (8, 9). Despite its prevalence, the majority of live traps in use are neither inspected nor certified prior to deployment (10).

To address concerns about humane trapping, the Agreement on International Humane Trapping Standards (AIHTS) was adopted in 1998 and entered into force in 2008 in only few countries, so that ratification remains limited. The AIHTS defines welfare indicators based on behavioral and injury-related criteria, alongside guidelines for trap inspection and approval. Technical specifications for mammal traps, including classification systems and testing methodologies, are set forth in the International Organization for Standardization ISO/TC 191, which, while influential, are not legally binding. Germany’s Animal Welfare Act, which has been anchored in the Basic Law since 2002, articulates the legal and ethical responsibility of humans toward animals (TSchG, 2006). The Act prohibits the infliction of pain, suffering, or harm on animals without reasonable cause and mandates that vertebrate killing be conducted with minimal unavoidable pain, requiring appropriate qualifications and competence (11).

Catching wild animals carries risks, including physical injury and physiological stress (4, 12, 13). Studies have demonstrated that live trapping may alter body parameters (14, 15) and cause tissue damage (14, 16). For coypu, research has addressed the efficiency of trapping and the effects of various management strategies (17, 18), including comparisons between individual and group trapping (19, 20), as well as bait preferences (21, 22). For raccoons, studies have evaluated different trap types regarding injury outcomes (23, 24). In some countries, “Egg traps” continue to be favoured over wire mesh traps (25–27). Evaluations of the “Krefeld Fox Trap,” a box trap, revealed paw and nasal injuries (28, 29). A broader overview of injuries caused by box traps in raccoons shows that most individuals experienced no or only minor trauma (10).

Despite extensive research, a universally accepted definition of animal welfare remains elusive (30). Historically, the” Five Freedoms” model has been the dominant framework focusing on the prevention of negative states in farm animals (31, 32). This model has since evolved into the more comprehensive “Five Domains” model, which incorporates nutrition, environment, health, and behavioral and mental state as key components of welfare (33–35). The Five Domains approach now guides welfare assessment in zoological and conservation settings as well (36, 37).

To assess physical trauma from trapping, researchers have developed trauma scoring systems based on pathological findings (10). These scales, refined over decades, form the foundation of the ISO 10990-5 guideline, which categorizes injuries into four severity levels: mild, moderate, moderately severe and severe.

According to the AIHTS, a live trap is deemed compliant if, among a sample of at least 20 individuals of the target species, 80% exhibit no indicators of reduced welfare. Section 2.3.2 of the standard (38) lists specific parameters (a-n) considered relevant indicators of animal welfare (Supplementary material).

Part of our study relates to the documentation of injuries sustained by the captured animals in connection with live capture and the assessment of the carcasses regarding the general health status and injuries sustained. The methodology is aligned with AIHTS and ISO10990-5 criteria, supplemented by additional protocols. Among the range of trap types described in the literature (3, 39), we focused on the three most commonly used box traps for trapping coypus or raccoons in Lower Saxony, Germany. This paper addresses the somatic condition of the captured animals; behavioral outcomes associated with live trapping are reported in a separate study (40).

## 2 Materials and methods

### 2.1 Study area and setup

From 2019 to 2023, a total of 55 coypus and 45 raccoons were captured using three different types of live traps in Lower Saxony, Germany. All captures were conducted in accordance with the applicable legal hunting seasons as stipulated by regional hunting regulations. The live trapping of animals was carried out as part of a research project approved by the Lower Saxony State Office for Consumer Protection and Food Safety (LAVES) (33.8-42502-04-19/3190). The project was conducted between 2019 to 2023 and was funded by the hunting levy of the Lower Saxony Ministry of Food, Agriculture and Consumer Protection. Additional carcasses were obtained between 2021 and 2023 from animals legally harvested with handguns by authorized hunters. Permission for these removals was obtained from the respective landowners or hunting leaseholders, and the carcasses were provided for research purposes.

**Figure 1:**
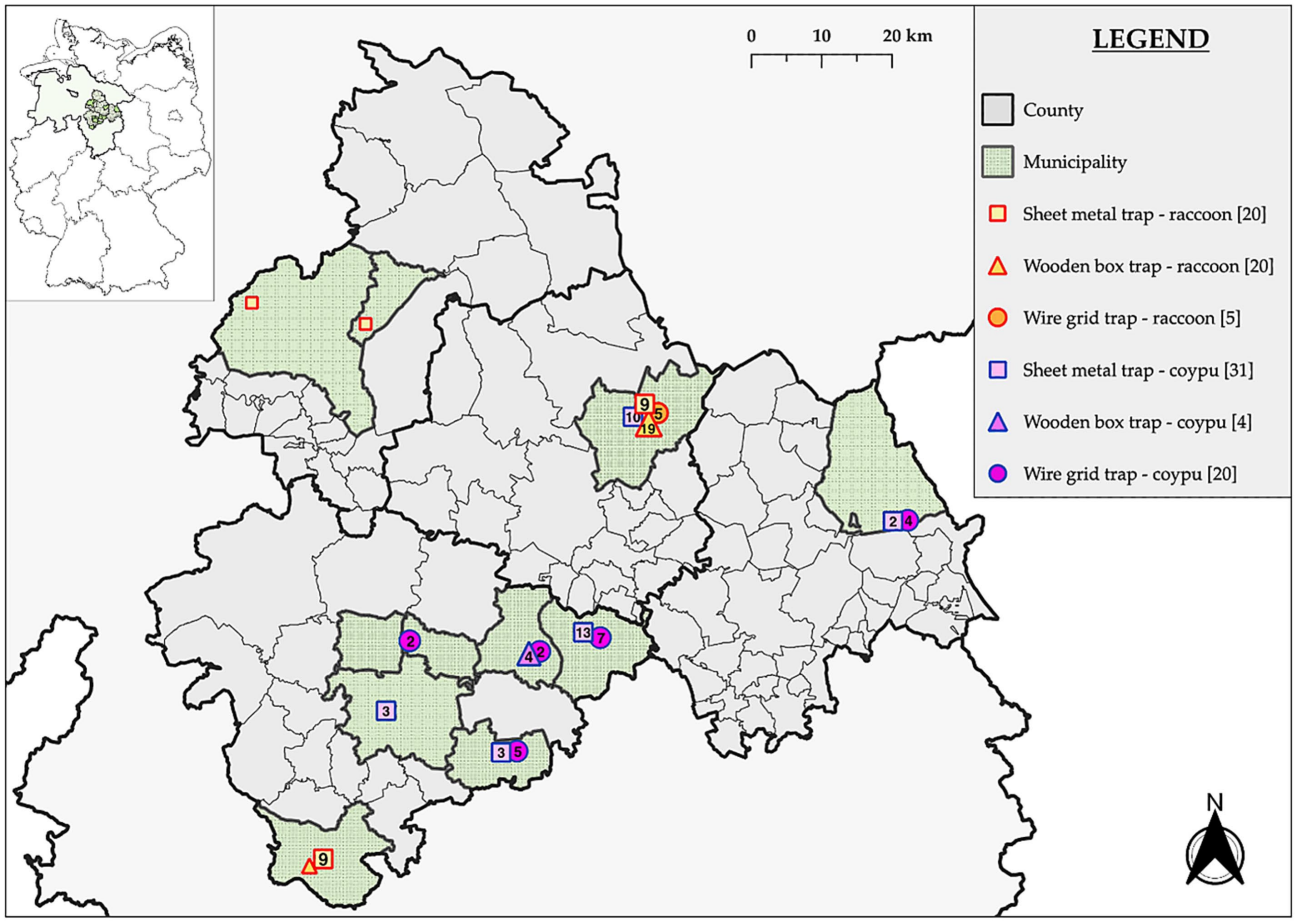
Locations of captures for coypus (purple, *n* = 55) and raccoons (orange, *n* = 45) conducted in the federal state of Lower Saxony, located in north-western Germany.

The experimental setup was designed to closely replicate practical field conditions and reflect standard trapping procedures used in regional wildlife management. Trapping sites were based on species presence and aligned with management plans developed by the Lower Saxony Chamber of Agriculture. The sites represented a range of environmental conditions, differing in vegetation structure, proximity to anthropogenic infrastructure, and broader landscape features. Locations were selected close to and based on previous evidence of tracks and camera traps of the respective animal species (see Figure 1).

**Figure 2:**
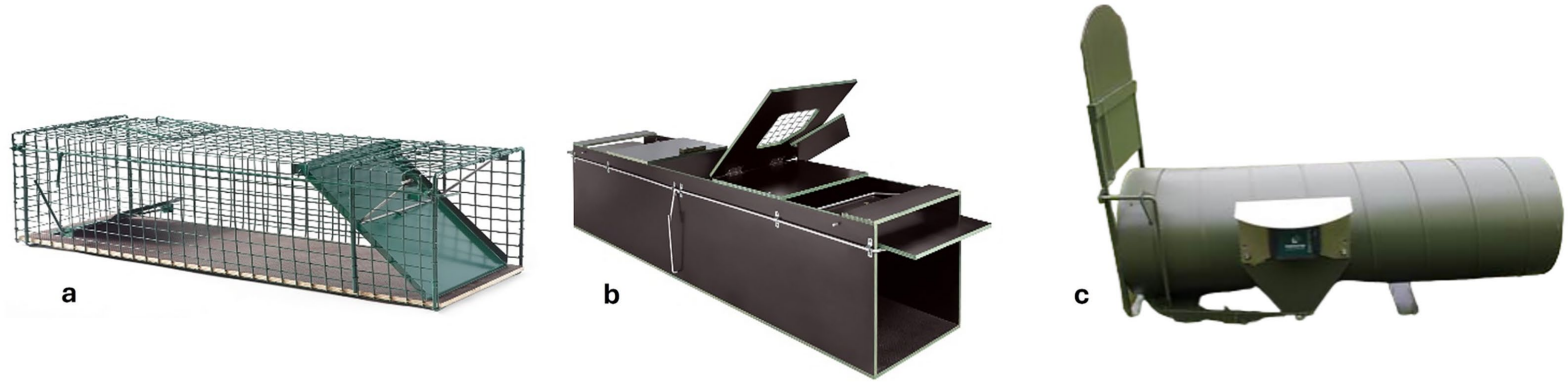
**(a)** WGT, with rocker board mechanism, Raimund Lorek^©^; **(b)** WBT, triggered by a rocker board or tripwire mechanism, Raimund Lorek^©^; **(c)** SMT “Trapper-Neozoen^®^,” with a tilting mechanism, Raiffeisen Warengenossenschaft Osnabrücker Land (RWO) eG^©^.

The distinct trap models commonly used in Lower Saxony were evaluated:

1. Wire grid trap (WGT, Figure 2a)

This model is a wire mesh trap typically used by water management associations. The trap measures 92×30.7×30.7 cm and features a 2.4 cm mesh size. It includes a bait basket positioned at the rear and is activated by a rocker mechanism located immediately in front of the basket. This type of trap was deployed without external covering.

2 Wooden box trap (WBT, Figure 2b)

Manufactured by fuchsfalle.de (Horb am Neckar, Germany), this trap consists of screen-printed panels measuring 150x31x34 cm. Two release mechanisms were tested with this trap type:

- A rockerboard release, consisting of a board reinforced by metal struts underneath, and
- A tripwire release, implemented as a transverse fishing line.

Both mechanisms are centrally mounted inside the trap. The doors close in a sloped fashion, and an inspection door is installed on the top to allow visual access to the interior.

3 The sheet metal trap (SMT, Figure 2c)

The “Trapper-Neozoen^®^” model (Raiffeisen Warengenossenschaft Osnabrücker Land eG, Melle, Germany) is composed of a galvanized steel tube mounted on a central support axis (dimensions: 106 × 36 × 63 cm). This trap operates by tilting when the animal’s weight shifts the centre of gravity, thereby releasing a drop-down door that fully seals the entrance. Notably, the trap contains no internal mechanical parts. Ventilation is provided by a series of small holes located at the rear of the trap.

All traps used during the experimental phase were equipped with electronic monitoring systems to ensure a comprehensive data collection. These systems included motion-triggered photo cameras, audio recorders, temperature sensors, and trap detection modules (TRAPMASTER professional^®^ and TRAPMASTER Neo^®^ EPV Electronics GmbH, Lüdenscheid, Germany), as described in Schöttes et al. (40). Only vegetarian bait (such as carrots, apple, and sweet scents) was used throughout the trapping period to standardize attractants and reduce variability in trapping success.

### 2.2 Sampling

After a maximum capture duration of six hours, coypus and raccoons captured during the animal experiment were transferred to a box, where anaesthesia was administered intramuscularly using medetomidine hydrochloride (Domitor^®^, Vetoquinol GmbH, Ismaning, Germany) and ketamine (Ketamine 10%, Ecuphar GmbH, Greifswald, Germany). Animals culled as part of routine hunting practice were either examined immediately after removal from the trap or stored at −20 °C and examined later.

Following anaesthesia, blood and hair samples were collected and vital parameters—including rectal body temperature, oxygen saturation, heart rate, and respiratory rate—were recorded during anaesthesia monitoring. Euthanasia was performed by intracardial administration of, T61^®^ (T61^®^, MSD Animal Health, Intervet Deutschland GmbH, Unterschleißheim, Germany).

Environmental parameters at the time of capture were documented, including ambient and internal trap temperature, weather conditions, trap location, trap type and exact trapping time.

A standardized carcass examination was performed postmortem following a protocol developed in consideration of ISO10990 (41, 42). Each carcass was weighed, and biometric measurements were recorded, including head-body length, head-tail length, head length and circumference, neck, thoracic and abdominal circumferences, height at withers, and hind foot length.

A comprehensive external examination assessed the general and nutritional condition, sex (43, 44) and age (juvenile <1 year/adult >1 year) based on dental status, development, and body weight (44–48). The external health assessment also included standardized inspection of the eyes, ears, nose, mouth, anus, genitalia, fur, skin, subcutaneous fat, and musculature. Standardized photographic documentation with special emphasis on injuries was completed for each individual, which focused on possible injuries and recorded the external health of the animal.

Radiographic examinations were conducted in-house at the Department of Small Mammal, Reptile and Avian Medicine and Surgery using standard imaging protocols. Thoracic, abdominal, cranial, and limb radiographs were acquired in right lateral, left lateral, and ventrodorsal views, using a Gierth HF400A X-ray unit (GIERTH X-Ray international GmbH, Riesa, Germany). The tube-to-film distance for overview images was 90 cm with settings of 44 kV and 6.81 mAs. For cranial and limb imaging, the distance was reduced to 70 cm, and settings adjusted to 44 kV and 4.48 mAs. Carcasses were placed directly on the detector plate without an X-ray grid. Depending on availability, CR MM3.0 Imaging digital imaging plates (Agfa HealthCare GmbH, Bonn, Germany) were used with the Digitizer CR-35-X (Agfa HealthCare GmbH, Bonn, Germany), or the FDR D-EVO II C35i detector (FUJIFILM Europe GmbH, Ratingen, Germany) was used. Thoracic and abdominal radiographs were taken in right and left lateral, as well as ventrodorsal views. Skull images were obtained in right or left lateral, dorsoventral, and angled projections with a mouth wedge. Paws of fore- and hindlimbs were imaged separately in dorsoventral, ventrodorsal and laterolateral orientations, ensuring parallel positioning. Based on the clinical examinations and X-ray, the age of the animals was estimated and classified as juvenile or adult, by body measurements, weight, sexual status (mature or immature), and growth plates (presence or absence) in comparison with literature (44–47).

Pathological examinations were conducted by veterinarians from the Department of Pathology, following a standardized protocol (41, 42). All organ systems were assessed macroscopically. The following organs were routinely collected for histopathological examination: femoral and/or sternal medulla, lymph nodes, skeletal muscle, diaphragm, tongue, tonsils, nasal skin, trachea, esophagus, heart, lung, spleen, liver, pancreas, kidneys, urinary bladder, stomach, small and large intestines, thyroid and parathyroid glands, adrenal glands, brain (frontal, parietal, temporal cortex, hippocampus, thalamus, cerebellum, brain stem), pituitary gland, sciatic nerve, spinal cord, and thymus. Tissue samples were fixed in 10% neutral buffered formalin, embedded in paraffin wax, sectioned at 3 μm thickness, and stained with hematoxilin and eosin (H&E) for histological evaluation.

Special stains – including Turnbull blue, Congo red, Ziehl-Neelsen, periodic acid-Schiff reaction (PAS)—were applied in selected cases. Virological (immunofluorescence) and parasitological (native smear) diagnostics were carried out at the Institute of Parasitology, focusing on the detection of intestinal parasites and, in raccoons, rabies virus (Rabies virus).

### 2.3 Data preparation and analysis

Data were compiled and processed using Microsoft Excel (Microsoft Excel Office 2016, Microsoft Excel Corporation, Redmond, WA, United States). Age classification of the animals (juvenile or adult) was based on a combination of external examination and radiographic findings, including body measurements, weight, sex status and the presence or absence of growth plates, and was performed in reference to published literature cited above. To improve model performance, we categorized the individual data on body weight, rectal temperature, and serum cortisol concentration, as well as the external variables for indoor and outdoor temperature, into classes, based on the median split (49) (Supplementary material). Time information was documented in Central European time, with seasonal and daily classification included. Weather conditions at the time of capture were characterized using standard meteorological parameters such as humidity, wind, cloud cover, precipitation and visibility (50).

The physical health status and observed injuries were coded using a predefined key to ensure consistent data categorization. This coding scheme supported both descriptive and statistical analyses. An etiological classification was applied to differentiate between injuries associated with trapping and those resulting from other causes. Injuries not attributable to the trapping process were included in descriptive analyses but excluded from statistical modelling of trap-related injuries. Injuries incurred during the transfer from the trap to the capture box, as well as agonal changes, post-mortem artifacts, and terminal events unrelated to trapping, were excluded from evaluation.

To assess the severity of trap-related injuries, a dedicated Injury Score (IS) was developed (Table 1). This scoring system was adapted from Byrne et al. (4), which itself builds on the simplified framework of Murphy (51) with modifications tailored to the present study. In addition to qualitative assessments, injury dimensions (in centimetres), as proposed by Van Ballenberghe (2), were incorporated to provide a quantitative measure of trauma. The IS comprises six categories reflecting increasing injury severity, “no injury,” “mild,” “moderate,” “severe,” “fracture,” and “death.” Each category includes symptom-based definitions, describing the type and extent of tissue affected. The score was compared with injury thresholds defined by the Agreement of International Humane Trapping Standards (AIHTS). Only scores 4 (“fracture”) and 5 (“death”) aligned with AIHTS criteria relevant to animal welfare.

**Table 1:** Injury score: the assessment of injuries incorporated quantitative and qualitative measure of trauma resulting in a categorization as an injury score.

IS	Classification	Symptoms	AIHTS relevant
0	No injury	No injury attributable to the trapping process	x
1	Minor injuries	Superficial skin lesion measuring 0–0.5 cm in length (single, localized areas, may be associated with slight subcutaneous hemorrhage or edema) Mild erythema or skin irritation Minor abrasion of hair or claw horn	x
2	Moderate injuries	Superficial skin lesion measuring 0.5–2.0 cm in length (single, localized areas). Tooth fracture involving less than half of the crown without exposure of the pulp cavity. Moderate abrasion of claw horn.	x
3	Severe injuries	Skin lesion exceeding 2.0 cm in length, involving either a single more extensive area or deeper tissue layers (potentially accompanied by more pronounced hemorrhage or edema). Tooth fracture involving more than half of the crown without exposure of the pulp cavity. Severe abrasion of claw horn.	x
4	Fractures	Deep, extensive lesions involving underlying structures such as bone or joints, with possible damage to tendons or joint capsules. Tooth fracture with exposure of the pulp cavity.	✓
5	Death	Death as direct result of trapping-related injuries.	✓

On the right column, relevance of the detected injuries in AIHTS is cross-referenced.

To evaluate the overall condition of each animal, an Overall Score (OS) was applied, adapted from Hubert et al. (24). This score reflects the most severe injury observed in an individual and assigns the animal to one of five severity categories. The classification was based on the injury considered most significant for the animals’ welfare.

### 2.4 Predictors

For the modelling of behavioral and injury-related datasets, independent variables were categorized into three groups:

1. Individual data: sex, age, and weight class.
2. External environmental variables: trap type, ambient temperature, season, and time of day.
3. Stress-related physiological parameters: rectal body temperature, internal trap temperature, and serum cortisol concentration.

The OS was used as the dependent variable in the model. In addition, the relationship between the anatomical localization of trap-related injuries (skin and soft tissue, claws, teeth, bones) and the type of trap used was analysed. For raccoons specifically, a separate analysis was conducted to examine the influence of the trap’s trigger mechanism on the OS.

### 2.5 Statistical analysis

Descriptive statistics were compiled using Microsoft Excel^®^ (Microsoft Excel Office 2016, Microsoft Excel Corporation, Redmond, WA, United States) and inferential statistical analyses were conducted using SAS Enterprise Guide 7.1^®^ (69).

A significance level of 5% (*α* = 0.05) was applied throughout. Tests for normality (Kolmogorov–Smirnov test: *p*-value < 0.010; Shapiro–Wilk test: *p* > 0.003) indicated that the data were not normally distributed (70). Homogeneity of variances was assessed using Levene’s test, with results supporting the null hypothesis of equal variances (H0 = 0.19). Logistic regression models were used to examine associations between predictor variables and the overall score, with OS serving as the dependent variable.

## 3 Results

### 3.1 Specification of dataset characteristics

Datasets from 55 coypus and 45 raccoons were included in the analysis (Tables 2–4).

**Table 2:** Individual data of coypus (*n* = 55) and raccoons (*n* = 45), including sex ratio, age, and weight classes.

Individual data		*N*	
		Coypu	Raccoon
Sex	Male	24	25
	Female	31	20
Age	Adult	31	17
	Juvenile	24	28
Weight [kg] Weight class	Light	19	15
	Medium	18	14
	Heavy	18	14
	NA	0	2

NA, data not available. *N* indicates the number of individuals sampled.

**Table 3:** External environmental variables recorded for coypus (*n* = 55) and raccoons (*n* = 45) including trap type, ambient temperature, season, and time of day.

External factors	Total			*N*	
	Coypu	Raccoon		Coypu	Raccoon
Trap type	55	45	WGT	20	5
			SMT	31	20
			WBT	4	20
Temperature outside [°C] Tout	51	23				T0	18	6	T1	16	7	T2	17	10	NA	4	22
			Season (meteorological)	55	45				Spring	13	4	Summer	0	22	Autumn	11	15	Winter	31	4
						Time of day (Central European time)	55	45				Morning	2	0	Before noon	0	1	Noon	1	0	Afternoon	3	0	Evening	25	7	Night	24	37

NA, data not available. *N* indicates the number of individuals sampled.

**Table 4:** Stress-related physiological parameters detected from coypus (*n* = 55) and raccoons (*n* = 45) including the rectal body temperature (Trec), internal trap temperature (Ttrap), and serum cortisol concentration (SCortisol).

Stress-related parameters	Total *N*			*N*	
	Coypu	Raccoon		Coypu	Raccoon
Rectal body temperature [C°] Trec	49	18			
			Low	17	6
			Medium	16	7
			High	16	5
			NA	6	27
Temperature trap inside [C°] Ttrap	25	0			
			Ttrap0	8	0
			Ttrap1	9	0
			Ttrap2	8	0
			NA	30	45
Serum cortisol concentration [nmol/l] SCortisol	50	18			
			SC1	14	5
			SC2	15	7
			SC3	21	6
			NA	5	27

NA, data not available. *N* indicates the number of individuals sampled.

According to the AIHTS, a minimum of 20 animals per trap type is required for valid trap performance evaluation. This threshold was met for coypus in the WGT (*n* = 20) and the SMT (*n* = 31), but not for the WBT (*n* = 4).

For raccoons, sufficient sample sizes were obtained for the SMT trap (*n* = 20) and the WGT (*n* = 20), whereas the WGT was underrepresented (*n* = 5).

To optimize triggering reliability, the WBT used for raccoon captures was modified midway through the study: the original tilting board release mechanism (*n* = 11) was replaced with a tripwire release (*n* = 9).

### 3.2 General health status

The majority of animals was assessed to be in good nutritional condition, including 38 coypus and 42 raccoons. A moderate nutritional status was recorded in 15 coypus (27%) and three raccoons (0.1%), while poor nutritional condition was identified in two coypus (0.04%) and one raccoon (0.02%).

Among the female coypus, ten individuals (32%) were found to be pregnant, carrying between two and ten fetuses at varying gestational stages. No pregnant raccoons were captured during the study period; however, one female exhibited a swollen mammary gland, indicating possible lactation.

Pre-existing pathological findings unrelated to trapping were observed in 91.0% of coypus (50/55) and 91.1% of raccoons (41/45). Approximately 9.0% of individuals in both species showed no detectable pre-existing lesions.

### 3.3 Pathological findings

In 54.5% of coypus (30/55) and 93.3% of raccoons (42/45), trap-related injuries were documented (Table 5), while 45.5% of coypus (25/55) and 6.7% of raccoons (3/45), showed no lesions that could be associated with capture.

**Table 5:** Trap-related injuries in coypus (*n* = 30) and raccoons (*n* = 42).

Organic system	Organ	Findings	*N*	
			Coypu	Raccoon
Skin and subcutis	Skin		**28**	**36**
		Alopecia	1	6
		Swelling (edema)	1	2
		Abrasion	20	34
		Gingival injury	3	1
		Superficial laceration	5	7
		Disruption of connective tissue	2	0
Musculoskeletal system			**4**	**39**
	Claw	Claw horn wear	2	39
		Haemorrhage	0	5
		Claw avulsion or tearing	2	0
	Bone	Fracture	2	0
Upper digestive tract			**6**	**6**
	Teeth	Dental trauma	1	6
		Tooth wear	5	0

The affected organ systems include the skin and subcutis, the musculoskeletal system, and the upper digestive tract. The findings for individual organs are based on macroscopic and histological examinations. *N* indicates the number of individuals sampled. Numbers in bold represent the total number of individuals with findings in the respective organ system.

#### 3.3.1 Localization and pattern of trap-related injuries

**Figure 3:**
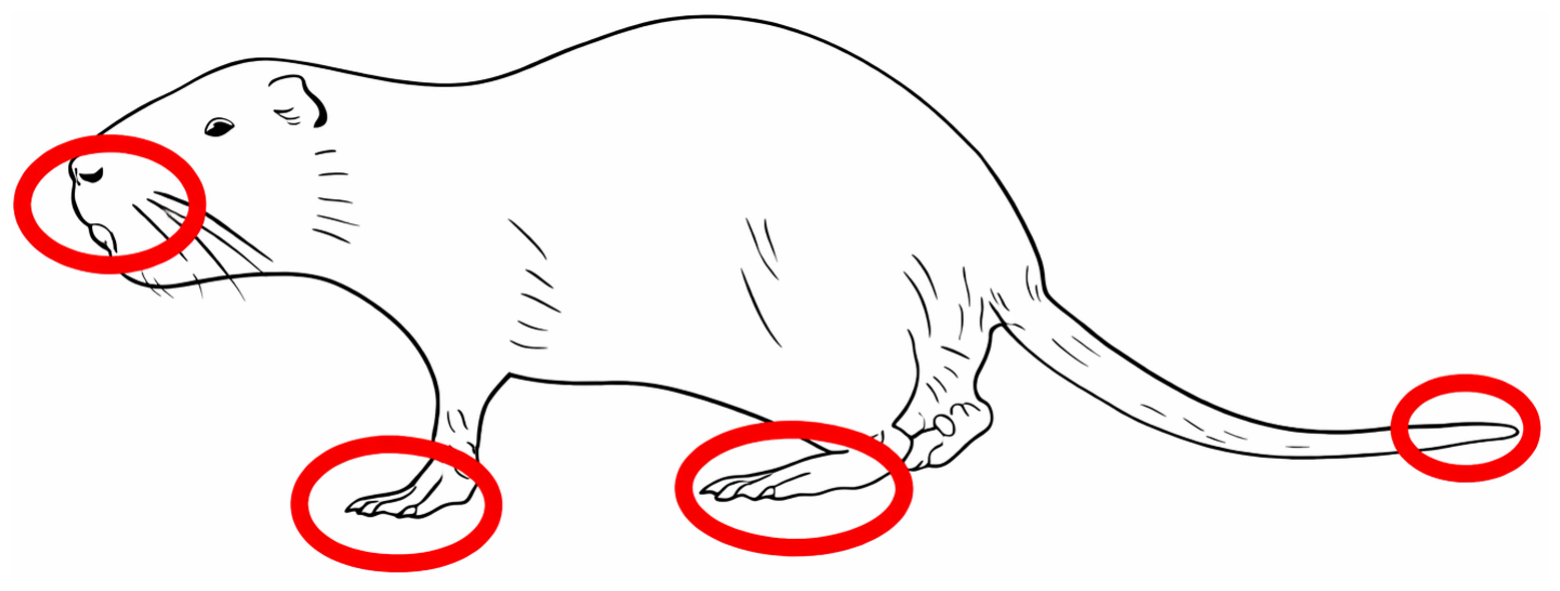
Predisposition of trap-related injuries in coypu, schematic illustration. Image rights: kindly provided with permission by C. Maistrelli.

In coypus (c), injuries to the skin and soft tissues that could be clearly attributed to the trapping process were predominantly located on the limbs, tail, and the oro-nasal region, including the lips, gums, and nasal bridge (Figure 3). The majority (71%) of skin lesions were classified as abrasions (*n* = 20), followed by superficial cuttings (*n* = 5). Fractures were detected in two individuals, one at the second digit of a forelimb and the second at an alveolar cavity with associated tooth loss. Claw injuries were most commonly observed on the hind limbs (1/4), whereas dental trauma primarily involved the upper incisors (4/6). Notably, 5 coypus showed injury patterns that were associated with skin and teeth lesions.

**Figure 4:**
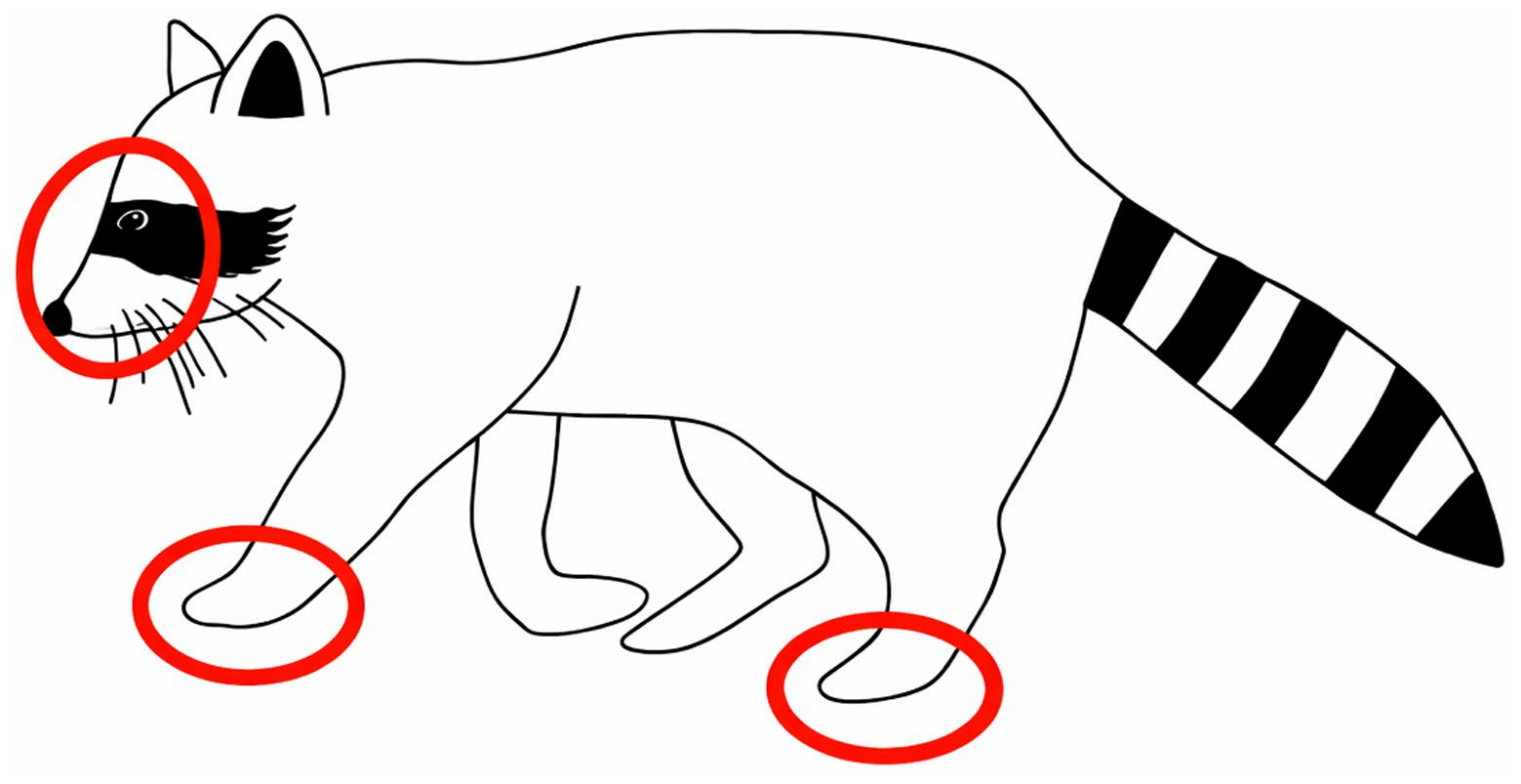
Predisposition of trap-related injuries in raccoons, schematic illustration. Image rights: kindly provided with permission by S. Schöttes.

In raccoons (r) (Figure 4), skin and soft tissue injuries were frequently noted on the limbs, particularly on the dorsal aspect of the forepaws, as well as on the snout and forehead. Claw injuries were mainly confirmed on the forelimbs, while dental injuries most often affected the canines and premolars or both the upper and lower jaws. The majority of raccoons showed a pattern of combined lesions located at skin and teeth (*n* = 28).

#### 3.3.2 Severity of trap-related injuries

**Figure 5:**
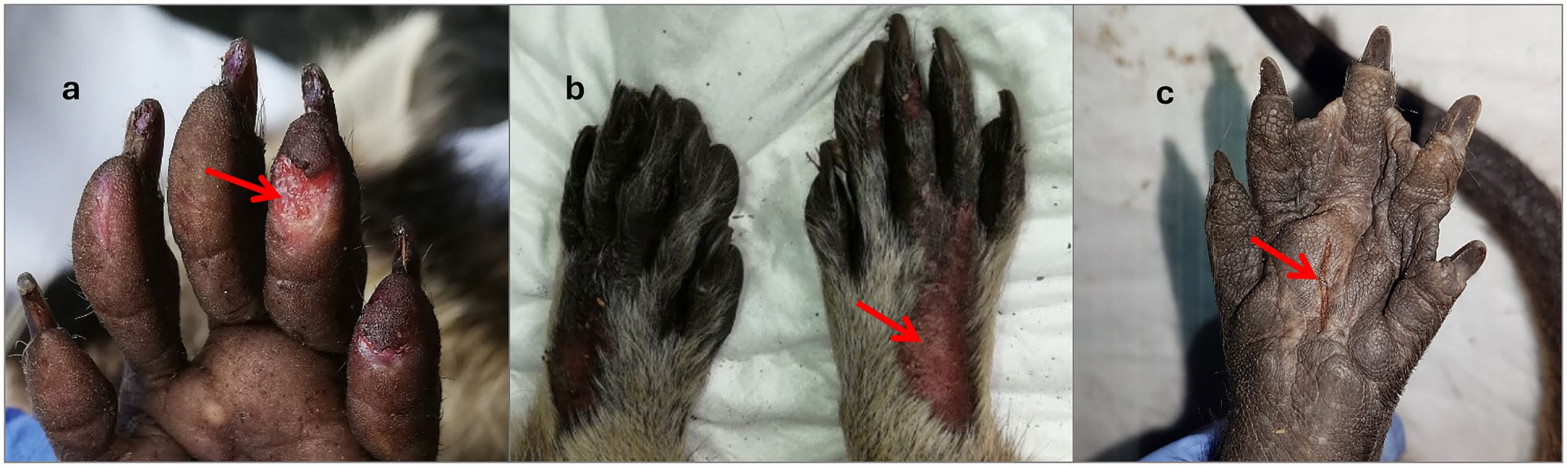
Examples of catch-related injuries on limbs: **(a)** Palmar cut on the second digit of the forepaw and worn claw horn of a raccoon corresponding to IS 2; **(b)** epithelial loss on the dorsal side of the forepaws with worn claw horn of a raccoon corresponding to IS 3; **(c)** laceration on the sole of a coypu’s hind limb, corresponding to IS 2.

**Figure 6:**
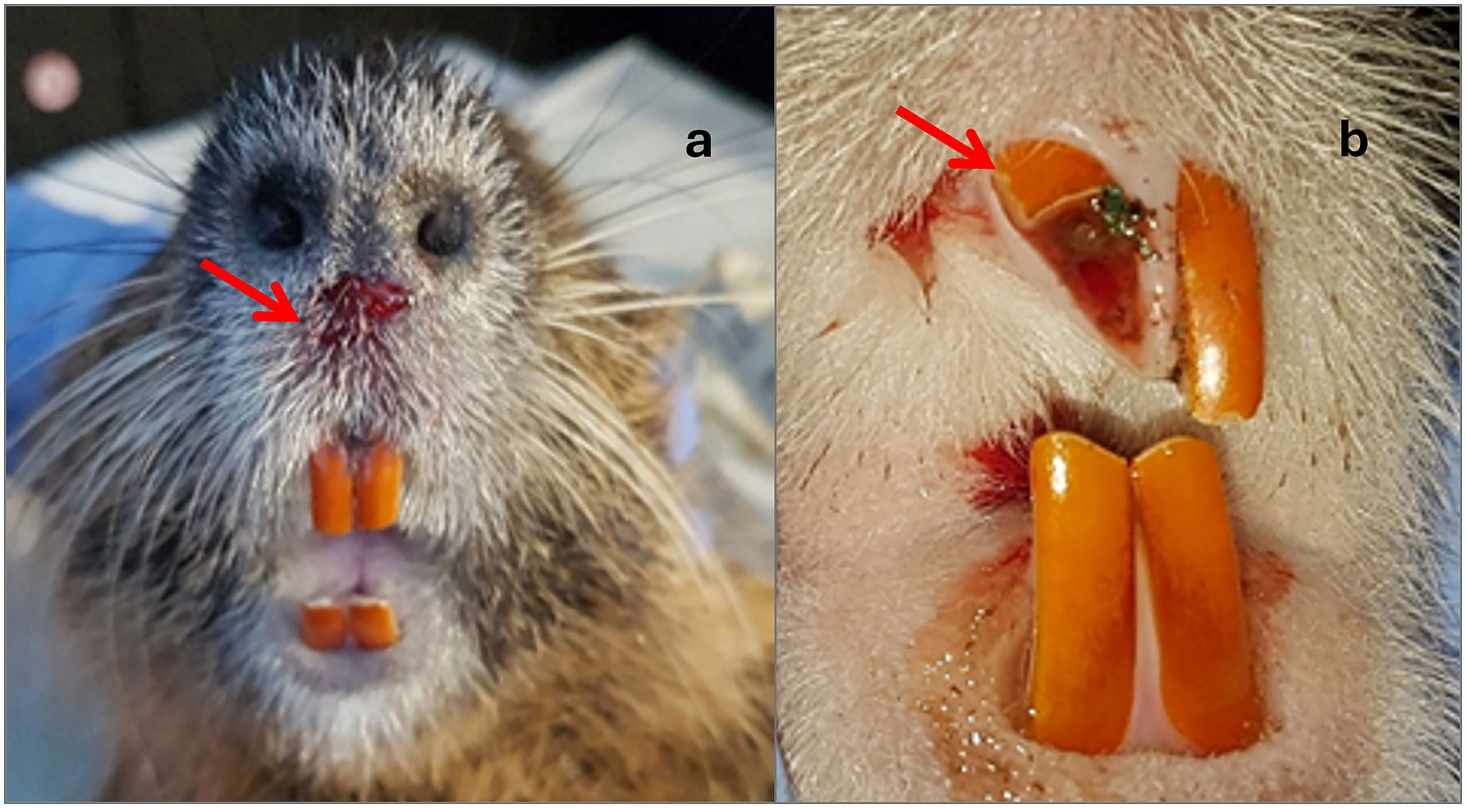
**(a)** Minor abrasion of the skin on the nasal surface of a coypu, **(b)** fracture of the upper third of an incisor in the maxilla of a nutria as a catch-related injury corresponding to IS 4.

**Figure 7:**
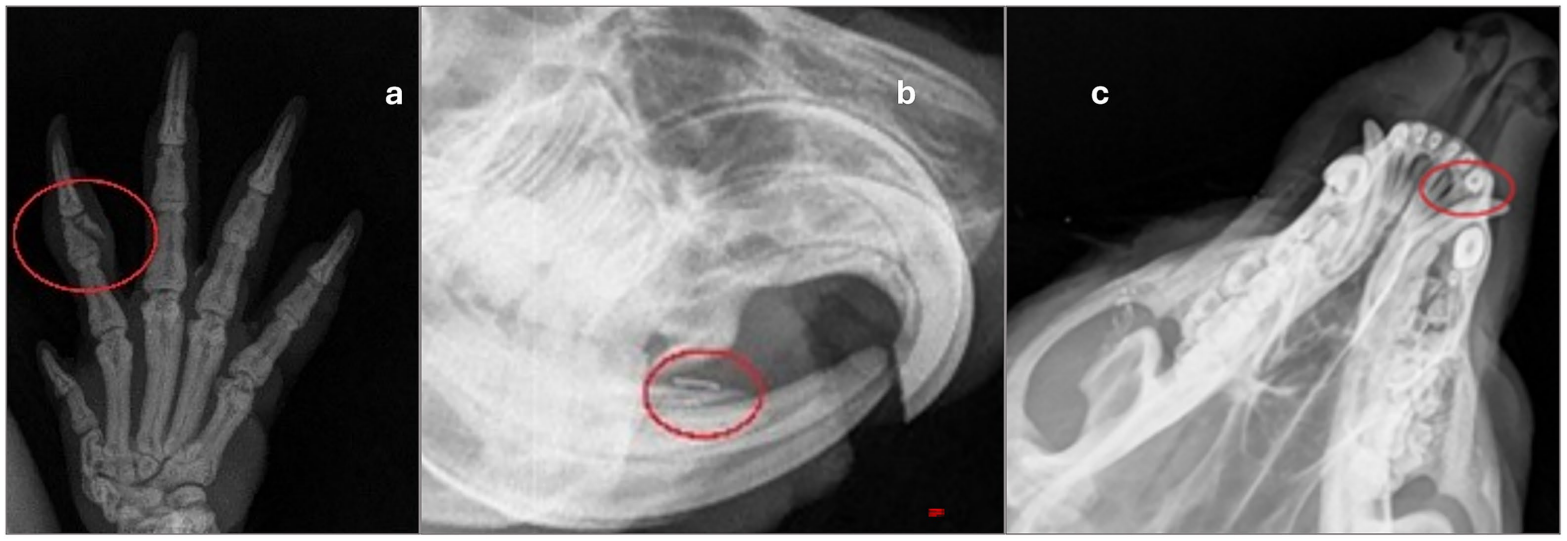
X-ray-findings of capture-related injuries corresponding to IS 4. **(a)** Fracture of the medial phalanx of the *digitus manus* of the middle right forelimb, with soft tissue swelling on the ventrodorsal radiograph of the right coypu forelimb. **(b)** In the right lower jaw, a splintered alveolus or mandible (without callus formation) is seen on a left-facing x-ray of a coypu skull. **(c)** Apical loss of substance of the I3, right side of the upper jaw of a raccoon, dorsoventral x-ray.

Trap-related injuries ranged in severity from minor to severe (Table 6). Trap-related trauma included hair loss (c: 1, r: 6), mild focal tissue swelling (c: 1, r: 1), and diffuse soft tissue swelling (r: 1). Superficial lacerations of the skin, with mild to moderate severity, occurred in five coypus and seven raccoons (Figures 5a–c). Furthermore, mild to moderate ulcerative abrasions of the skin epithelium, accompanied by fibrin extravasation and superficial hemorrhage, were observed in 20 coypus and 34 raccoons (Figure 6). Two coypus exhibited severe ulcerative tissue separation involving both skin and muscle at the tail, accompanied by focal hemorrhages in the hind limbs. In addition to dental trauma, minor gingival lesions were detected in three coypus and one raccoon. Claw injuries varied in severity, from mild to severe abrasions of the claw horn (c: 2, r: 39), to subungual haemorrhage in five raccoons. Claw avulsion was noted in two coypus. A fracture of the third phalanx of the second digit on the forelimb was identified in one coypu (Figure 7a), and another coypu sustained a mandibular fracture with a concurrent tooth fracture, including exposure of the pulp cavity (Figure 7b). Dental injuries were also documented: dentin wear in five coypus and chipping or breakage of tooth structures in one coypu (Figure 6b) and six raccoons. In three raccoons, pulp cavity involvement in fractured teeth could not be conclusively assessed; in one case, a pulp injury was diagnosed (Figure 7c).

**Table 6:** OS of coypus (*n* = 55) and raccoons (*n* = 45), in context to the single catch-associated lesions (IS).

OS	IS	*N*	
		Coypu	Raccoon
0	No injury	25	3
1	Minor injuries	15	8
2	Moderate injuries	10	20
3	Severe injuries	3	11
4	Fractures	2	3
5	death	0	0

*N* indicates the number of individuals sampled.

#### 3.3.3 Impact on trap-related injuries

In live-trapped coypus and raccoons, injuries were observed; however, no significant associations were identified between the occurrence or severity of these injuries and the individual data (e.g., age, weight class, sex), external environmental factors (e.g., season, time of day, trap type, outside temperature), or stress-related physiological parameters (e.g., serum cortisol concentration, rectal body temperature, internal trap temperature) as modelled in this study (Table 7). Four separate models were used, each calculated using logistic regression (Type 3 effect analysis). The results indicated no statistically significant association between the tested variables and OS, as reflected by the *p*-values from the chi-square test (Pr > ChiSq) (Table 7).

**Table 7:** Results for potentially influencing parameters on the OS of coypus and raccoons.

Model	Variables	Pr > ChiSq		*N*	
		Coypu	Raccoon	Coypu	Raccoon
Individual data	Age	0.6964	0.1540	55	43
	Sex	0.2945	0.3071		
	Weight class	0.3544	0.6295		
External factors	Trap type	0.1298	0.8618	51	23
	Season	0.1804	0.9991		
	Daytime	0.1372	0.8754		
	Temperature outside	0.2286	0.2346		
Stress-related parameters	Rectal body temperature	0.0723	0.3967	22	15
	Trap temperature	0.4155	na		
	Serum cortisol	0.2499	0.1740		
Trigger mechanism		NA	0.2841	NA	20

NA—data not available due to missing occurrence. *N* indicates the number of individuals sampled.

Regarding the trigger mechanism of the SD trap, which was modified during the study period, no statistically significant difference in frequency or severity was detected in the raccoon dataset between the two release variants (Table 7). For coypus, only the rocking board trigger mechanism was applied throughout the entire study period.

No significant influence of trap type on the occurrence of skin and soft tissue injuries in either species, nor on claw injuries in raccoons, could be demonstrated (Table 8). The model outcomes related to the effect of trap type on the incidence of dental and skeletal injuries in both species, as well as on claw injuries in coypus, must be interpreted with caution due to the limited sample size and the resulting restricted validity of these analyses.

**Table 8:** Model outputs for trap type-association with locations of injuries in coypus and raccoons, based on a logistic regression (type 3 effect analysis).

Model	Variables	*N*		Pr > ChiSq	
		Coypu	Raccoon	Coypu	Raccoon
Trap type	Skin and soft tissue	28	36	0.9930	0.1140
	Tooth	6	6	0.9073	0.8095
	Claw	4	39	0.9956	0.1766
	Bones	2	0	0.9978	NA

NA—data not available due to missing occurrence. *N* indicates the number of individuals sampled.

## 4 Discussion

Although this study employed a comprehensive range of investigative methods, the limitation of the trapping period to six hours represents a constraint for assessing animal welfare impacts. This timeframe does not reflect the typical duration for which traps are deployed in practice. Standardized assessment schemes adopted from laboratory animal research are only partially applicable to wild animals, as the latter often present with pre-existing injuries or physical impairments. These differences necessitate a reconsideration of what constitutes an “intact” baseline. In contrast to wild animals, laboratory animals can be examined in vivo without prior injuries and are typically habituated to human handling.

This analysis, as part of a broader study investigating the live trapping of coypus and raccoons using three different trap types, focused on the injuries sustained by the animals during the trapping process. Wild animals often conceal signs of distress or injury, even when experiencing significant pain (52). As a result, external indicators alone are often insufficient for assessing stress. Therefore, parameters that reflect the impact of trap capture on the animals are used for evaluation. Thus, physiological and behavorial parameters are employed to evaluate the effects of trapping. Reported physiological indicators include body temperature, heart and respiratory rates, and levels of serum cortisol, muscle enzymes, bilirubin, or neutrophil counts (7, 15, 16, 53–55). Behavioral alterations—such as increased or decreased activity, digging, movement patterns, or gnawing at trap parts—also serve as important stress indicators (16, 56).

Injury localization is influenced by species-specific coping strategies (e.g., escape attempts, searching behavior, passive waiting) as described by Wechsler (57). These behaviors, combined with anatomical and behavioral traits, may result in distinct injury patterns between species (58). In this study coypus predominantly interacted with their environment using their teeth and snout, making these areas prone to injury. Of six documented dental injuries in coypus, five involved mild dentin wear, and only one showed an incisor fracture. In raccoons, six dental injuries were observed, including fractures of canines and premolars. The characteristic orange coloration of coypu incisors is due to iron deposition, which may increase resistance to breakage and result in wear rather than fractures under stress. Raccoons, on the other hand, have omnivorous dentition with pointed canines, which are more susceptible to fractures under mechanical strain.

Raccoons also exhibit highly manipulative, paw-oriented behaviors – traits they share with mustelids – which explains the prevalence of injuries to their forelimbs. This observation aligns with findings from previous studies (28, 29).

Regarding species differences, raccoons demonstrated higher activity levels in traps, leading to more severe injuries and higher overall scores (OS), while coypus sustained more minor injuries and thus received lower OS values. The musculoskeletal system was predominantly affected in raccoons, likely due to claw use during escape attempts. Raccoons frequently showed claw horn wear and haemorrhage (c: 4, r: 39), while coypus, presented claw fractures (c: 2, r: 0), reflecting species-specific anatomical and behavioral differences.

Trap-related injuries, including skin abrasions and broken teeth, have been documented in other studies using box traps or cage traps, typically occurring with lower frequency and severity compared to other trap types (3, 59). Box traps are generally associated with reduced physiological stress and are considered the least harmful among live traps (10, 15, 60). Iossa et al. provided a comprehensive review of injury data across species. One report indicated that 52% of raccoons captured in box traps sustained no injuries (10), a figure significantly higher than the 6.7% (3/45) observed in this study.

To effectively reduce injuries, it is essential to investigate the factors influencing an animal’s reaction to capture. While individual, physiological factors as well as external parameters appear influential, statistical significance could not be demonstrated in this study due to limited sample sizes. Nevertheless, observable differences between trap types and species-specific responses suggest meaningful trends.

The trap design plays a critical role in determining the animal’s stress level and injury profile. Structural elements influence where and how the animal attempts to escape. For example, in the WBT, light entering through the trap door slit may lead animals to target that area for escape, increasing the risk of injury. The trigger mechanism also presents potential hazards, as evidenced by injuries resulting from the broad, movable trigger platform of the WBT. These included typical injuries to the dorsal and palmar aspects of the raccoon’s front paws and damage to their canines. In contrast, the SMT- a round model with minimal internal structure—offers fewer attack points but can cause claw wear due to repeated contact with its smooth, hard surfaces. One coypu in the SMT exhibited a circular tail lesion, likely from entrapment in the trap door, possibly triggered by a previously captured animal. The open-sided WGT does not inherently minimize potential points of interaction with the outside. In this traptype, animals are more focused on their environment; with coypus showing increased digging behavior, and raccoons frequently extending their forelimbs through the sides. This is consistent with behavioral observations from other open trap types (23) and support the view that trap configuration strongly influences how animals respond, and therefore, the types and severity of injuries incurred.

When the results are analyzed by trap type, it becomes evident that injury severity and behavioral stress indicators must be interpreted in combination. In the WGT, animals exhibited high activity levels and alertness (40), but not necessarily a higher number or severity of injuries. Environmental shielding – such as trap covers – can reduce external stimuli and associated distress (71). Conversely, the WBT, with its sound-dampening wooden construction, was associated with more severe raccoon injuries. These contrasting findings suggest that no single indicator – whether behavioral or physiological – provides a sufficient measure of stress. Comprehensive multiparameter assessments are required. Therefore, trap type selection should be tailored to the target species, local conditions and construction features.

Beyond trap type, other internal and external factors—including age, sex, weight, weather and environmental context—could reveal significant associations with injury patterns if analyzed across larger datasets, as shown in a study of live-captured European badgers (4). Most commercially available live traps have not undergone formal testing for animal welfare or injury prevention. A list of current test procedures was compiled for the German Federal Environment Agency (61). However, formal certification remains rare, and most evaluations emphasize technical functionality over animal welfare considerations. Some injuries likely predated the trapping event, emphasizing that wild animals often arrive in compromised physical condition. This underscores the importance of contextualizing observed injuries within the broader framework of life in the wild.

Proulx et al. (62) offer a robust framework for best practices in live trapping, encompassing trap placement, timing, monitoring, animal handling, and operational training. Every field application, including live trapping, must be carefully evaluated for necessity and executed with diligence and preparation (1, 5, 62, 63). A strong ethical commitment to animal welfare is essential for maintaining public support, as societal awareness of animal well-being and wild animal protection continues to grow (64, 65). Consequently, transparent, rigorous, and science-based implementation of live trapping protocols is essential for the long-term success of wildlife management programs (66).

Assessing the severity and evaluating associated pain, suffering, and stress remains a complex challenge. A thorough understanding of species-specific sensory and nociceptive capacities is essential to adequately evaluate pain perception and stress intensity (67). However, objective, quantifiable metrics for assessing emotional and physiological stress in animals remain underdeveloped (67), and further research in this area is urgently needed.

## 5 Conclusion

Necropsies of 55 coypus and 45 raccoons demonstrated that trap-related lesions occurred in 54.5% of the coypus (30/55) and 93.3% of the raccoons (42/45). These findings underscore the influence of trap design on the type and severity of injuries sustained during live capture. To improve animal welfare outcomes, technical aspects of trap construction must be critically evaluated and optimized. This includes selecting appropriate materials based on environmental conditions (e.g., season, temperature) and ensuring that the interior surfaces are free of protrusions or movable components that could pose injury risks or serve as focal points for escape attempts. Moreover, minimizing the duration of confinement is essential. The use of trap alert systems with automated reminder functions is recommended to enable prompt monitoring and timely intervention. Taken together, these measures can substantially reduce stress and physical trauma in captured wildlife and support more ethically and scientifically sound trapping practices.

## Data Availability

The original contributions presented in the study are included in the article/Supplementary material, further inquiries can be directed to the corresponding author.
