# Supplementary material for: Trap-related injuries in coypus (Myocastor coypus) and raccoons (Procyon lotor)—an approach to improve animal welfare in live trapping
Source: Front Vet Sci. 2026 Jan 23;12:1752107. doi: 10.3389/fvets.2025.1752107 (PMC12880112; doi:10.3389/fvets.2025.1752107)
Supplement: Supplementary file 1 [file Data_Sheet_1.PDF]

Supplementary Material

- # Trap-related injuries in coypus (*Myocastor coypus*) and raccoons (*Procyon lotor*) - an approach to improve animal welfare in live trapping

Friederike Gethöffer[1†], Franziska Schöttes[1†], Maximilian Reuschel[2], Peter Wohlsein[3], Andreas Beineke[3], Ursula Siebert[1*]

[1]Institute for Terrestrial and Aquatic Wildlife Research, University of Veterinary Medicine Hannover, Foundation, Bischofsholer Damm 15, 30173 Hannover, Germany

[2] Department of Small Mammal, Reptile and Avian Medicine and Surgery, University of Veterinary Medicine Hannover, Foundation, Bünteweg 9, 30559 Hannover, Germany

[3] Department of Pathology, University of Veterinary Medicine Hannover, Foundation, Bünteweg 17, 30559 Hannover, Germany

1. Excerpt of Rule Number: 42 1997 Off. J. Eur. Communities by the European Community, and Government of the Russian Federation,

Page 47 – Indicators,

2.3.2. Inquiries recognised as indicators of poor welfare in trapped wild animals are:

(a) fracture;

(b) joint luxation proximal to the carpus or tarsus;

(c) severance of a tendon or ligament;

(d) major periosteal abraison;

(e) severe external haemorrhage or haemorrhage into an internal cavity;

(f) major skeletal muscle degeneration;

(g) limb ischaemia;

(h) fracture of a permanent tooth exposing pulp cavity;

(i) ocular damage including corneal laceration;

(j) spinal cord injury;

(k) severe internal organ damage;

(l) myocardial degeneration;

(m) amputation;

(n) death.

2. Excerpt of Rule Number: 42 1997 Off. J. Eur. Communities by the European Community, and Government of the Russian Federation,

Supplementary Table 2.1 Predictor Categories

2.1. **The table presents the predictors included in the logistic regression models for coypus and raccoons. Three categories were defined: individual data, external factors, and stress-related parameters. All variables are of categorical type.**

**Model**	**predictor**	**variable**	**description**
**Individual data**	gender	male<br>female	
	age	juvenile<br>adult	< one year<br>> one year
	weight class	light<br>medium<br>heavy	coypu: 0.8-2.0 kg<br>raccoon: 1.5-3.52 kg<br>coypu: 2.1-4.8 kg<br>raccoon: 3.52-6.0 kg<br>coypu: 4.9-6.8 kg<br>raccoon: 6.0-8.4 kg
**External factors**	trap type	wire grid (WGT)<br>sheet metal (SMT)<br>wooden box (WBT)	open trap type<br>closed trap type<br>closed trap type
	season	spring<br>summer<br>autumn<br>winter	01.03.-31.05.<br>01.06.-31.08.<br>01.09.-30.11.<br>01.12.-29.02.

	time of day (CET)	morning<br>before noon<br>noon<br>afternoon<br>evening<br>night	06:00:00-09:59:59<br>10:00:00-11:59:59<br>12:00:00-13:59:59<br>14:00:00-16: 59:59<br>17:00:00-20:59:59<br>21:00:00-05:59:59
	temperature outside (Tout)	Tout0<br>Tout1<br>Tout2	-1.0-3.0 C°<br>3.1-7.0 C°<br>7.1-18.6 C°
**Stress-related parameters**	rectal body temperature (Trec)	low<br>medium<br>high	coypu: 32.1-33.5 C°<br>raccoon: 36.5-38.17 C°<br>coypu: 33.6-35.0 C°<br>raccoon: 38.17-38.9 C°<br>coypu: 35.1-38.81 C°<br>raccoon: 38.9-41.1 C°
	temperature trap inside (Ttrap)	Ttrap1<br>Ttrap2<br>Ttrap3	-0.38-4.4 C°<br>4.5-8.4C°<br>8.5-24.0C°
	Serum Cortisol (SCortisol) [nmol/l]	SC1<br>SC2<br>SC3	40.80- 395.71<br>395.72-621,67<br>621.68-1086.96
